# Epigenetic modulation of brain gene networks for cocaine and alcohol abuse

**DOI:** 10.3389/fnins.2015.00176

**Published:** 2015-05-20

**Authors:** Sean P. Farris, Robert A. Harris, Igor Ponomarev

**Affiliations:** Waggoner Center for Alcohol & Addiction Research and The College of Pharmacy, University of Texas at AustinAustin, TX, USA

**Keywords:** addiction, cocaine, alcohol, chromatin, gene co-expression networks

## Abstract

Cocaine and alcohol are two substances of abuse that prominently affect the central nervous system (CNS). Repeated exposure to cocaine and alcohol leads to longstanding changes in gene expression, and subsequent functional CNS plasticity, throughout multiple brain regions. Epigenetic modifications of histones are one proposed mechanism guiding these enduring changes to the transcriptome. Characterizing the large number of available biological relationships as network models can reveal unexpected biochemical relationships. Clustering analysis of variation from whole-genome sequencing of gene expression (RNA-Seq) and histone H3 lysine 4 trimethylation (H3K4me3) events (ChIP-Seq) revealed the underlying structure of the transcriptional and epigenomic landscape within hippocampal postmortem brain tissue of drug abusers and control cases. Distinct sets of interrelated networks for cocaine and alcohol abuse were determined for each abusive substance. The network approach identified subsets of functionally related genes that are regulated in agreement with H3K4me3 changes, suggesting cause and effect relationships between this epigenetic mark and gene expression. Gene expression networks consisted of recognized substrates for addiction, such as the dopamine- and cAMP-regulated neuronal phosphoprotein *PPP1R1B/DARPP-32* and the vesicular glutamate transporter *SLC17A7/VGLUT1* as well as potentially novel molecular targets for substance abuse. Through a systems biology based approach our results illustrate the utility of integrating epigenetic and transcript expression to establish relevant biological networks in the human brain for addiction. Future work with laboratory models may clarify the functional relevance of these gene networks for cocaine and alcohol, and provide a framework for the development of medications for the treatment of addiction.

## Introduction

Addiction to alcohol and other drugs of abuse is a prevalent problem in our society, affecting millions of individuals worldwide. Approximately 50% of the risk for the development of addiction may be due to inherited genetic differences within hundreds or thousands of genes (Bierut, 2011). Genetic variation however may not fully account for the occurrence of substance dependence, and the biochemical changes involved in this mental health disorder. Acute, as well as repeated, administration of cocaine and alcohol, can lead to significant changes in gene expression throughout different areas of the brain (Lewohl et al., 2000; McClung and Nestler, 2003; Kerns et al., 2005; Piechota et al., 2010; Mulligan et al., 2011). Longstanding alterations in gene expression in response to chronic drug abuse are steered, at least in part, by epigenetic factors in the absence of DNA sequence variation (Maze and Nestler, 2011). Characterizing diverse epigenetic processes and their connection with global regulation of gene expression profiles may yield insights into the molecular basis of addiction.

Variation in the quantity of specific histone modifications may lead to chromatin-remodeling and promote downstream alterations in gene expression (Karlić et al., 2010). Trimethylation of one particular epigenetic marker, histone H3 at lysine 4 (H3K4me3), is highly enriched at transcription start sites and associated with active transcription (Strahl et al., 1999; Schübeler et al., 2004). The hippocampus is one of several key brain regions in the neurocircuitry of addiction (Koob and Volkow, 2009) and regulation of H3K4me3 in adult hippocampus has been linked to the formation of memory (Gupta et al., 2010), suggesting this epigenetic modification and following changes in gene expression may contribute markedly to CNS plasticity. Relative levels of H3K4me3 abundance and gene expression across the genome constitute highly orchestrated biological processes that are capable of contributing to habitual addictive behavior. We hypothesize that habitual cocaine and alcohol abuse leads to revision of the epigenetic and transcriptional landscape, causing interdependent changes to biological networks that are unique to each substance of abuse. To date, no effort has been made to define the relationships between epigenetic and transcriptional processes in drug addiction at the network level.

Charting the pairwise relationship of H3K4me3 abundance and gene expression activity can reveal systematic patterns governing cellular function. We used previously published high-throughput sequencing data (Zhou et al., 2011) to superimpose variation in chromatin-immunoprecipitated (ChIP-Seq) H3K4me3 bound DNA with variation in transcriptome (RNA-Seq) to expose coherent groups of genes burdened by long-term cocaine and alcohol exposure within human hippocampus. Distinguishing coordinately expressed groups of genes, declared as co-expression modules, places attention on interactions rather than potentially disparate sets of individual genes. Emergent physiological and behavioral phenotypes of substance abuse may not be tied directly together across the H3K4me3-transcriptome space, but arise as a result of coinciding gene clusters. Linking the genome-wide arrangement of signal levels for H3K4me3 with gene co-expression networks, our analysis revealed biologically plausible hippocampal gene networks for substance abuse. These networks outline a distinct fraction of genes modulated by either chronic cocaine or alcohol exposure via epigenetic changes. Although additional studies are needed to explain the functional importance of these gene networks, some of the genes residing within these networks have been previously associated with addiction. Perturbations to these critical genes may disseminate to neighboring genes throughout the network, causing pronounced effects on neurobiological systems within the hippocampus in a drug specific manner of action.

## Materials and methods

### Initial collection and data analysis

Collection of postmortem human brain tissue and initial processing of next-generation sequencing data has been previously described (Zhou et al., 2011). Log transformed and normalized RNA-Seq and ChIP-Seq data was kindly provided by Dr. David Goldman, Section Chief of Human Neurogenetics at National Institute for Alcohol Abuse and Alcoholism. Human samples from the Miami Brain Bank with matching RNA-Seq and ChIP-Seq datasets were selected for downstream analysis, resulting in a total of twenty-three hippocampi from eight alcohol abusers, seven cocaine addicts, and eight matched control subjects. Matching samples permitted a direct comparison of histone H3 lysine 4 trimethylation (H3K4me3) with gene expression across individuals. Cross-sample comparison demonstrated a mean inter-sample correlation equal to *r* = 0.90 (range: 0.80–0.94) for transcriptome studies, and an *r* = 0.85 (range: 0.73–0.91) for epigenome studies; indicating there were no discernable outliers. H3K4me3 peaks were annotated for gene symbols based upon the nearest transcription start site using the ChIPpeakAnno package in R (Pagès et al., 2010). Peak annotation per unique gene reduced the total number of peaks from 27,569 to 14,295 marks for further analyses.

Differential expression was determined on the total number of detected features within RNA-Seq and ChIP-Seq samples using linear modeling (LIMMA) (Ritchie et al., 2015). An uncorrected *p*-value threshold set at 0.05 for assessing statistical significance to minimize potential type II error, and retain a sufficient number of genes for conducting over-representation analyses of gene ontologies, canonical molecular pathways, and other gene network dynamics. Despite using a slightly different statistical method for the determination of differential expression and a different technique for matching H3K4me3 peaks with their respective gene targets, our LIMMA analysis captured 99.7% of the RNA-Seq and 77.9% of the ChIP-Seq results previously reported on these human hippocampal datasets (Zhou et al., 2011).

### Network analysis of RNA-Seq and ChIP-Seq data

**Figure 1:**
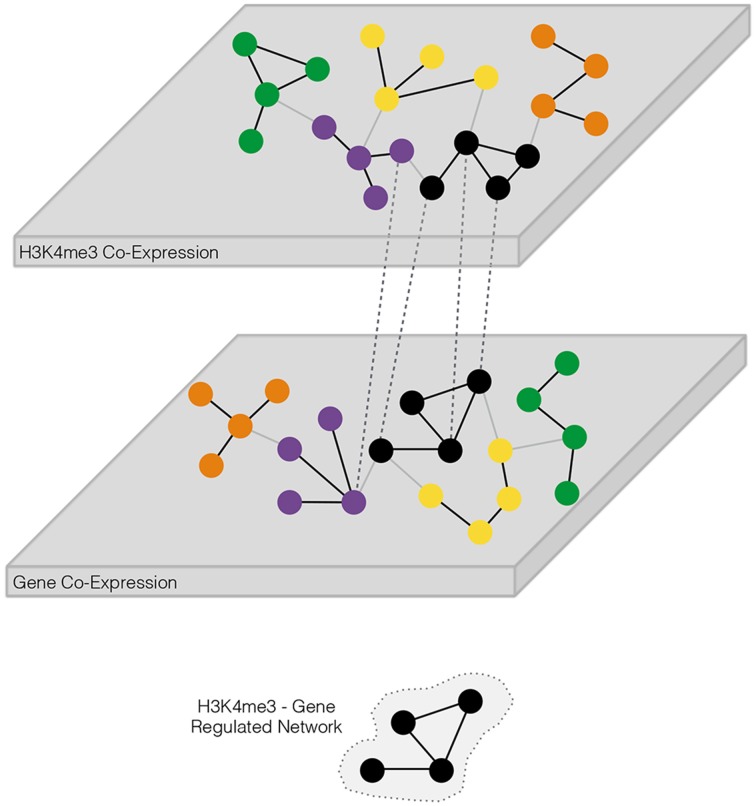
**Schematic overview of approach for identification of coordinately regulated trimethylation of histone H3 at lysine 4 (H3K4me3) and transcriptome gene networks**. The two “omics” techniques, representing different layers of biology, are separately analyzed for the detection of co-expressed targets. Coordinately regulated genes form biologically relevant groups known as modules. H3K4me3 and transcriptome networks are “stacked” to single-out those modules possessing a significant number of shared genes, defining an H3K4me3—gene co-expression regulatory network. Modules within each stack may be further tested for differentially expressed targets to determine if one or more substances of abuse perturb H3K4me3-gene co-expression regulatory network(s).

The primary focus of this manuscript is to characterize gene co-expression networks in the context of regulation through H3K4me3 for cocaine and alcohol addiction in postmortem human brain tissue. A schematic representation of our network approach is shown in Figure 1. After filtering for common identifiers between RNA-Seq and ChIP-Seq a total of 11,088 genes were remaining for further investigation. Weighted gene co-expression network analysis (WGCNA) was performed using the WGCNA package in R (Zhang and Horvath, 2005; Langfelder et al., 2008) separately for RNA-Seq and ChIP-Seq datasets. Four genes (*CALCA, MFAP2, MME, PLEK2*) were excluded due to a lack of variance across samples. Network construction of expression estimates for human postmortem samples has previously been described (Ponomarev et al., 2012; Farris et al., 2014). A signed network was built using a soft-thresholding power ß = 5 for RNA-Seq expression, and ß = 8 for ChIP-Seq expression to yield scale-free topology model fit *R*^2^-values equal to 0.90 for both datasets. Dendrograms, representing the inter-correlation among genes, were split into co-expression modules based upon the dynamic tree cut method (Langfelder et al., 2008) with a minimum module size of 100 and cutting height of 0.99 for both RNA-Seq and ChIP-Seq data. Signed WGCNA has multiple advantages over standard methods for differential expression analyses, including helping overcome issues related to multiple testing, for multi-dimensional data that incorporates genome-wide patterns of epigenetic modifications and gene expression (Mason et al., 2009). Functional enrichment was determined for modules using functions available through WGCNA (Langfelder and Horvath, 2008), centered upon the terms defined by the Gene Ontology Consortium (Ashburner et al., 2000). Depicted graphical models for H3K4me3-Gene module relationships, and gene co-expression relationships, were assembled using the open source software Cytoscape (Shannon et al., 2003; Cline et al., 2007). In addition, we evaluated the robustness of our gene co-expression networks by comparing them to previously published networks in superior frontal cortex (CTX) from human alcoholics (Ponomarev et al., 2012). Briefly, the gene overlap between all possible pairs of modules was estimated and the significance of module overlap was assessed using a hypergeometric test.

## Results

### Differential expression

**Figure 2:**
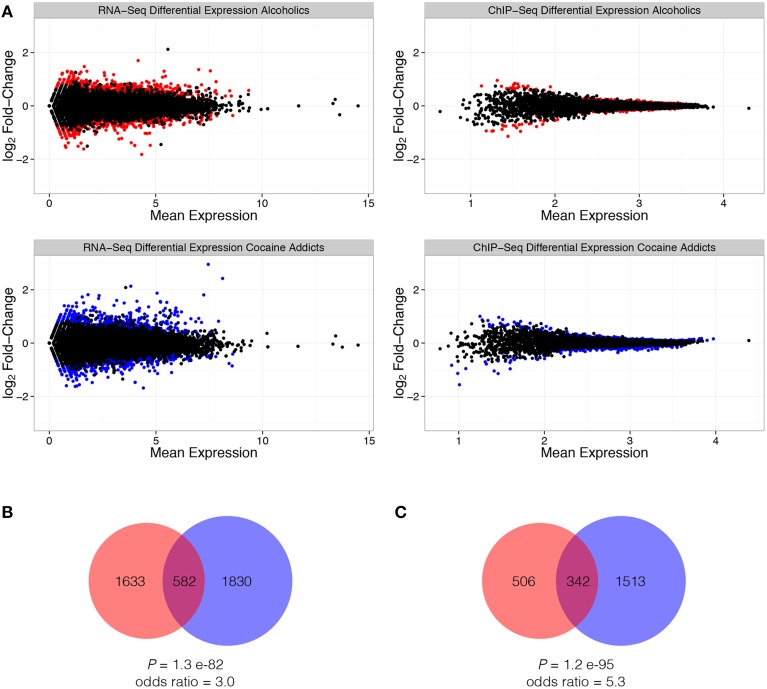
**Differential expression due to either alcohol or cocaine addiction from ChIP-Seq (H3K4me3) and RNA-Seq normalized expression values**. Scatter plots demonstrate mean expression vs. log_2_ fold-change in expression for RNA-Seq (left) and ChIP-Seq (right) differential expression within alcoholics (top) and cocaine addicts (bottom). Red data points indicate differentially expressed genes (*P* < 0.05) from alcoholics, while blue data points indicate differentially expressed genes (*P* < 0.05) from cocaine addicts. Solid black data points correspond to non-differentially expressed genes **(A)**. A significant number of transcriptome changes **(B)** and H3K4me3 changes **(C)** are shared between alcoholics (red) and cocaine addicts (blue).

Differentially expressed genes or transcripts targeted by histone H3 lysine 4 trimethylation (H3K4me3) may have important implications for addiction. A total of 2215 and 2412 genes are differentially expressed (*P* < 0.05) due to alcohol abuse or cocaine addiction, respectively (Supplemental Table 1). Alterations in H3K4me3 targeted expression were less robust, with 848 changes in alcoholics and 1855 changes as a result of cocaine addiction (Supplemental Table 2), but are consistent with previously reported results (Zhou et al., 2011). Fewer differences in direct targets of H3K4me3 vs. transcriptome measurements may be a reflection of the numerous additional molecular elements, such as separate histone modifications or transcription factors, regulating gene expression. Cocaine and alcohol addicted individuals shared a significant number of differentially expressed features for both RNA-Seq and ChIP-Seq (Figure 2). Commonalities between alterations due to either chronic cocaine or alcohol exposure could indicate shared neurobiological factors to these substances of abuse; however, the mutual list of genes does not necessarily indicate they are related to one another. Additionally, the identification of differentially expressed genes does not provide information as to the neighboring genes, and the molecular pathways, that may be subsequently affected as a result of chronic substance abuse. Weighted gene co-expression network analysis (WGCNA) was applied to construct molecular networks, which will assist in discerning the shared molecular targets influenced by chronic drug exposure.

### Network analyses

Gene network analyses provide a systems-level context of biological relationships based in part on the pairwise relationship of gene expression profiles. Functioning within a larger network, individual genes may cluster into distinct groups that are important for neuropsychiatric disorders and coordinating efforts across differing layers of molecular information. WGCNA respectively identified 25 and 28 modules for RNA-Seq and ChIP-Seq (Figure S1). Modules represent groups, or clusters, of strongly coexpressed (coregulated) genes within the hippocampus; including at least one module which is consistent with brain-region specific markers of postmortem human hippocampus (*P* = 3.66E-15) (Hawrylycz et al., 2012). Each of the identified clusters represent coordinately regulated genes that participate in numerous known biological processes, cellular components, and molecular functions (Supplemental Table 3). Portraying gene expression and H3K4me3 alterations as modules helps condense large amount of information into discernable units of biology. Although multiple factors may regulate gene expression, H3K4me3 in promoters is a prominent chromatin modification, which can mark actively transcribed genes (Barski et al., 2007; Guenther et al., 2007). Previous analysis of these data (Zhou et al., 2011) showed that H3K4me3 mark alone could account for no more than 10% of transcriptomic variance, suggesting that correlating ChIP-Seq and RNA-Seq data across the whole genome is not a very powerful approach and partitioning of variance may be required to identify robust correlating patterns.

**Figure 3:**
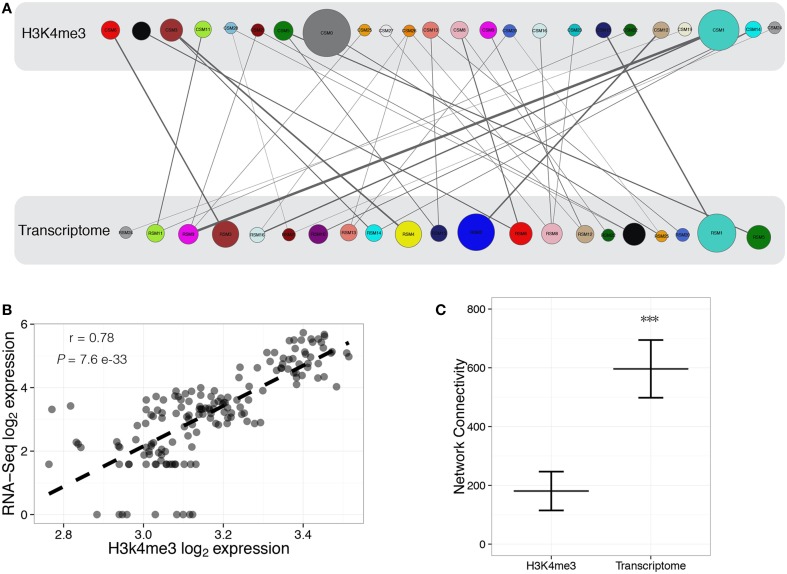
**Characterization of modules for H3K4me3 regulation of transcriptome dynamics**. Construction of H3K4me3-transcriptome regulatory bipartite network, where circles represent WGCNA defined modules for ChIP-Seq (CSM) and RNA-Seq (RSM). Biological groups are represented as colored circles, with color corresponding to the individual modules identified through WGCNA. The sizes of circles are proportional to the number of objects within the module, with the width of the connecting line proportional to the number of common objects between H3K4me3 (top) and transcriptome (bottom) modules **(A)**. Representative correlation (Pearson's *r* = 0.78, *P* = 7.6 e−33) between log_2_ expression of H3K4me3 data (CSM26) and log_2_expression of RNA-Seq data (RSM16) **(B)**. Total network connectivity is significantly (*P* < 2.0 e−16) different between H3K4m3- and transcriptome-defined networks **(C)**.

ChIP-Seq modules (CSM) were compared with RNA-Seq modules (RSM) to discern those modules that may be biologically regulated as a group by trimethylation of histone H3 lysine 4. There were 35 significant (*P* < 0.05) overlaps between the two datasets (Figure 3A, Supplemental Table 4), with 83% of modules having significant positive correlation between H3K4me3 and transcript abundance. One example is a correlation between a mutual subset of genes belonging to CSM26 and RSM16 across samples (Figure 3B). This implies coordinated regulation of several gene sets in parallel to H3K4me3 modification, suggesting a causative relationship between these events. The number of overall interconnections within the H3K4me3 network is reduced compared to the transcriptome (Figure 3C). Differences in network connectivity between this histone modification and the transcriptome demonstrates disparities among the degree of biologically related modalities between the two systems. Each biological layer, whether it be epigenomic or transcriptomic, may be arranged in a slightly different manner, but coexist within the framework of a system to carry out the needs of the local cellular environment. Association between subgroups of chromatin-DNA interactions and the transcriptome could serve as biological motifs that support additional chromatin modifications, regulatory elements, and neighboring genes. Chronic abuse of cocaine or alcohol, and other environmental stimuli, may cause subtle remodeling of multiple epigenetic marks within fully matured brain cells. Although our analysis focuses on only one histone mark in relation to gene expression, it presents a structured view of gene sets potentially modulated through H3K4me3-marked promoters that can be further investigated in relation to substance abuse.

### Disease-related modules

**Figure 4:**
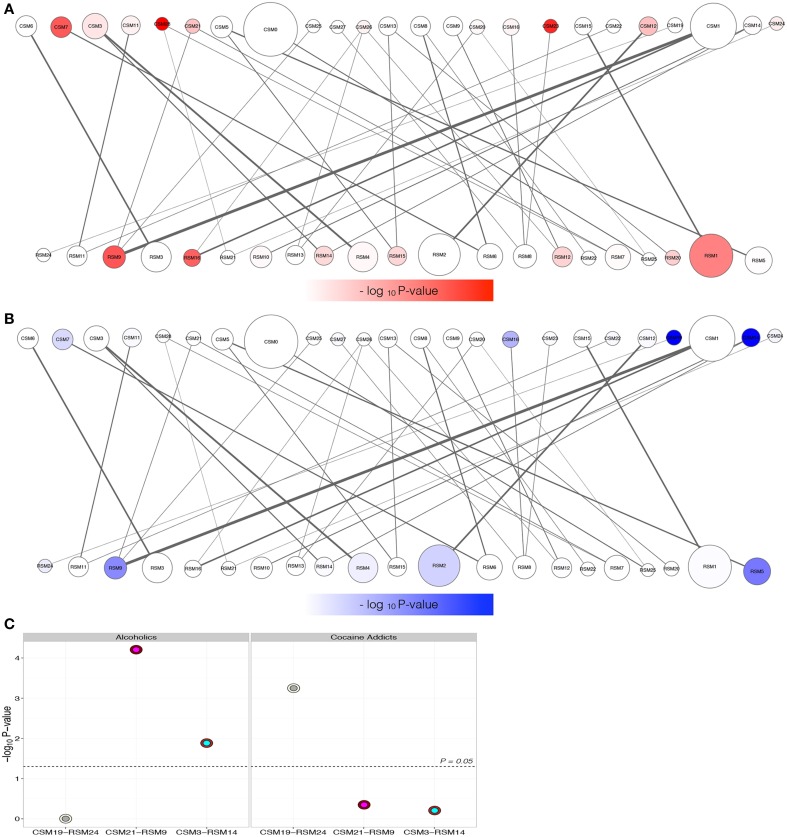
**Determination of modules for the H3K4me3-transcriptome regulatory bipartite network from Figure 3**. Modules enriched for differentially expressed genes due to either alcoholism **(A)** or cocaine addiction **(B)** within the H3K4me3 (top) or transcriptome networks (bottom). The degree of red (alcoholism) or blue (cocaine addiction) color corresponds to the relative –log_10_ *P*-value enrichment of differential expression within the respective groups. Only three coordinately targeted module pairs of ChIP-Seq (CSM) and RNA-Seq (RSM) are over-represented for differentially expressed targets according to disease status **(C)**. The x-axis plots the mean –log_10_ *P*-value determined using a hypergeometric test versus the CSM-RSM pairings (Figure 3A). Outer circles correspond to the CSM, with the inner circles corresponding to the partnering RSM. Alcoholics have 2 H3K4me3—gene co-expression regulatory module pairs (CSM21-RSM9 and CSM3-RSM14) enriched for differentially expressed targets, versus only one (CSM19-RSM24) for cocaine addiction.

**Figure 5:**
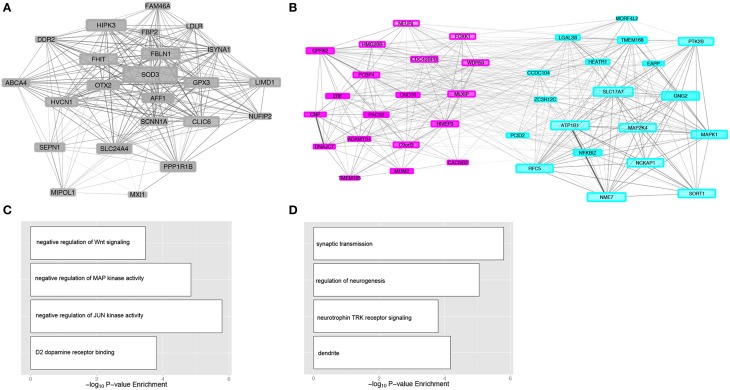
**Gene co-expression modules associated with substance abuse**. Network visualization of genes for cocaine addiction module RSM24 **(A)** and alcohol dependence modules RSM9 and RSM14 **(B)**. Connecting lines illustrate significant correlations (*P* < 0.05) between genes, scaled in size according to the strength of correlation. Shown are only hub genes from each module and genes with greater network connectivity are shown in larger sizes. Color of the gene networks coincide with respective RNA-Seq module assignments. Selected gene ontologies are shown for the cocaine module RSM24 **(C)** and alcohol dependence modules RSM9 and RSM14 **(D)** for categories related to CNS function.

Gene modules were overlaid with enrichment of differential expression due to either alcohol dependence (Figure 4A) or cocaine addiction (Figure 4B) to identify matching H3K4me3 and transcriptome modular differences related to disease status (Supplemental Table 5). Although more changes were detected in cocaine addicts than alcoholics (Figure 2), differentially expressed genes fell within a similar number of co-expression modules. There are 8 modules enriched for transcriptome changes and 7 modules enriched for H3K4me3-associated changes in alcoholics, while cocaine affected 7 modules within the transcriptome and 5 within H3K4me3. The limited number of modules affected suggests that differentially expressed attributes related to substance abuse are coordinately regulated, but are selectively spread throughout a few different biological groups. These disease-related modules represent biological processes, such as genes critically involved in synaptic transmission (RSM14, *P* = 2.73E-04) that may have not been identified without a network-based approach (Supplemental Table 3). Importantly, three mutually related CSM and RSM were enriched for differentially regulated genes related to chronic exposure of alcohol or cocaine (Figure 4C). The CSM-RSM pairs impacted were not identical for the two different drugs of abuse, demonstrating drug-specific mechanisms of action within human hippocampus. Chronic alcohol exposure altered expression of genes within CSM21-RSM9 and CSM3-RSM14 that have a combined 807 genes, while cocaine influenced genes within CSM19-RSM24 containing 133 genes. Networks of hub genes from gene co-expression modules are shown in Figure 5. Although not every individual gene within their respective CSM-RSM cohorts is altered by substance abuse, a significant fraction is impacted that will affect neighboring genes and overall neurobiological responses. This is in contrast to global assessment for differential gene expression because our analysis sought out to ascertain gene co-expression networks, downstream of H3K4me3 modifications, affected by substance abuse. Location of genes within co-expression networks can signify biological properties, which are important to the overall system. Clustering of these genes within a defined module represents a system that may facilitate the long-term effects of alcohol dependence within the hippocampus. Module affiliation of all matched genes is shown in Supplemental Table 6.

To validate our network approach, we compared gene co-expression modules from the present analysis to modules obtained from postmortem brains (CTX) of alcoholics and matched control cases using microarrays (Ponomarev et al., 2012). We identified overlapping genes between all possible pairs of modules from the two studies and calculated statistical significance of the overlap. Twenty three out of 25 RSM modules significantly overlapped with at least one CTX module from the 2012 study (Supplemental Table 7), validating the robustness of gene co-expression networks and suggesting conserved patterns of gene regulation in different brain regions of human alcoholics.

## Discussion

Epigenetic modifications of histones are capable of regulating long-lasting changes in gene expression. H3K4me3 is a promoter-enriched chromatin mark that may be critical for transcriptional activation (Schneider et al., 2004). Histone acetylation and methylation patterns, including those of H3K4me3, are not uniform throughout the human genome (Wang et al., 2008), leading to an assortment of gene activation and repression. Variation in chromatin states and gene expression can be interpreted as complementary biological networks fulfilling cellular demands in response to environmental stimuli. Repeated exposure to substances of abuse may lead to persistent addictive behavior as a result of coordinately regulated gene co-expression networks within discrete areas of the brain. The hippocampus is one of several vulnerable brain regions involved in addiction, which is responsible for short- and long-term memory processes (Nestler, 2002). Our analysis used a network-based approach centered on the H3K4me3 epigenomic and RNA transcriptional landscape to understand potentially key molecular maladaptations taking place within the hippocampus of humans following chronic exposure to cocaine or alcohol. The present study determined that variation in H3K4me3 abundance reliably predicts transcriptional changes for subsets of functionally related genes in the hippocampus of drug abusers. Using the network approach we validated some previous findings and provided an integrated view of brain changes associated with drug addiction.

Epigenetic factors, such as H3K4me3, are a proposed mechanism in the regulation of stable changes to gene expression and subsequent functional plasticity causing persistent addictive behavior (Robison and Nestler, 2011). Through a systems-based approach our analysis delineates gene expression networks affiliated with genome-wide patterns of H3K4 trimethylation for cocaine and alcohol abuse. Moving beyond gene-centric approaches that emphasize the biological role of a single candidate gene in addiction toward gene-network based approaches helps link together multiple genes that may collectively operate in driving addiction process. Genes correlated in their expression patterns as a network are often biologically related, participating in a common cellular pathway or acting within the same cell-type. The expression of gene networks within the human brain is reproducible across individuals (Hawrylycz et al., 2012; Konopka et al., 2012; Ponomarev et al., 2012) and our analysis validated this finding. Although addiction to different substances of abuse may converge on some common genes, some mechanistic networks acted upon may be different between differing brain regions and differing drugs of abuse.

Cocaine and alcohol addiction resulted in a number of differentially regulated features, with a significant number of shared changes occurring in response to these two drugs of abuse. Concentrating on networks of coordinately expressed genes and H3K4me3 binding targets we were able to ascertain biologically relevant modules separately responding to cocaine and alcohol abuse. We identified three epigenome-transcriptome pairs of modules that were affected by either cocaine or alcohol and hypothesized that the coordinated regulation of genes by chronic drug abuse may be specifically due in part to H3K4me3 epigenetic control of disease status. Comparison of our results to previously published gene networks in alcoholic brain (Ponomarev et al., 2012) showed that alcohol-regulated modules RSM9 and RSM14 overlapped with several CTX modules that were also alcohol-responsive, with the majority of genes in these modules being regulated in the same direction in both studies. Strikingly, several genes from the CTX modules were associated with the regulation of H3K4me3 in their promoters (Ponomarev et al., 2012), which is consistent with our finding of strong associations between the two RSM modules and corresponding H3K4me3-driven CSM networks. These data validate, at least in part, our network approach to identify epigenetic components critical for regulation of gene expression in disease. Some other modules besides RSM24, RSM9, and RSM14 were significantly over-represented for differentially expressed genes due to cocaine and alcohol abuse, but were not aligned with H3K4me3 modules. In agreement with the previous report, cocaine selectively affected a coordinately expressed group of genes (RSM2) related to the mitochondrial inner membrane and oxidative phosphorylation (Supplemental Table 3) (Zhou et al., 2011); however, these changes do not significantly coincide with H3K4me3 changes related to chronic cocaine exposure. Characterizing differential gene expression networks in light of alternative histone H3 modifications, as well as additional histones, could further our understanding of regulatory domains and pathways transformed in drug addiction.

One clear advantage of the network approach is prioritization of differentially regulated genes for follow-up studies. Because statistical significance does not necessarily imply functional significance, focus of genomic studies that use systems approaches is shifted from most statistically significant candidates to hub genes in drug-responsive biologically relevant networks. This approach highlights genes with some known involvement for addiction, but it may also help discern novel hippocampal transcripts for alcohol and cocaine addiction. For example, the modules for cocaine (RSM24) and alcohol (RSM9) addiction each contained an intracellular chloride ion channel. *CLIC6* was part of the cocaine-responsive network, while *CLIC4* was part of the alcohol-responsive network. CLIC6 may interact with dopamine receptors (Griffon, 2003), potentially affecting dopaminergic responses to cocaine. Manipulation of expression for the intracellular chloride channel gene *Clic4* is able to alter behavioral responses to alcohol across species (Bhandari et al., 2012), suggesting it may be an important target in acute and long-term effects of alcohol. Relative imbalance in the number of drug-responsive modules, and numbers of genes within those modules, between cocaine and alcohol abuse may be due in part to the more restricted pharmacological effects of cocaine on dopaminergic processes through competitive inhibition of the dopamine transporter (Ritz et al., 1987); however, the pharmacological effects of alcohol are exerted by a wide-array of molecular mechanisms (Harris et al., 2008; Ron and Messing, 2013). In general agreement with prior evidence of cocaine acting upon the dopaminergic system (Kuhar et al., 1991; Nader et al., 2006; Volkow et al., 2011), the cocaine-responsive module RSM24 was significantly enriched for molecules capable of acting as dopamine (DRD2) receptor binding proteins (*P* = 1.56E-4). One noteworthy member of RSM24 is protein phosphatase 1 regulatory subunit 1B (*PPP1R1B*), also known as dopamine- and cAMP-regulated neuronal phosphatase (*DARPP-32*). *PPP1R1B* encodes for a key phosphoprotein involved in the regulation of several signaling cascades for dopaminoceptive neurons across several areas of the brain (Fienberg et al., 1998; Greengard et al., 1999; Svenningsson et al., 2004), which is also required for the biochemical effects of cocaine (Zachariou et al., 2006). Mitogen-activated protein kinase family members *MAP2K4* and *MAPK1* were two of the highest-ranking hub genes of RSM14. In addition to relaying cellular signals, MAPK1 (also commonly referred to as ERK2 or p38) may confer DNA-binding activity (Hu et al., 2009) and selectively phosphorylate histone H3 following exposure to alcohol and the alcohol metabolite acetaldehyde (Lee and Shukla, 2007). Glutamate, a major excitatory neurotransmitter system implicated in alcohol dependence (Tsai and Coyle, 1998), can also induce phosphorylation of histone H3 through activation of MAPK1 (Brami-Cherrier et al., 2007). RSM14 was enriched for genomic clustering of genes related to glutamatergic neuronal function (*P* = 4.09E-10), including the top-ranking hub gene vesicular glutamate transporter *SLC17A7*.

Epigenetic adaptations causing changes in the expression of transcriptional networks could also be acting indirectly through microRNAs, small non-coding RNAs spread throughout the genome that are also known to regulate gene expression (Sato et al., 2011). Psychiatric disorders may arise through cooperation of numerous epigenetic processes and microRNAs, which affect the regulation of gene within both mature and newly forming hippocampal cells (Hsieh and Eisch, 2010). MicroRNA miR-9 is one example of a small non-coding RNA that post-transcriptionally regulates the expression of specifics genes to influence alcohol tolerance (Pietrzykowski et al., 2008). Among thousands of microRNAs potentially involved in the neurobiology of disease miR-9 is brain-specific (Sempere et al., 2004; Farh et al., 2005) with family members of this particular microRNA are among the highest ranked targets of H3K4me3 within neural progenitor cells (Benayoun et al., 2014). Although our analysis does not directly address a potential role for H3K4me3-microRNA regulation of gene expression networks, the gene expression modules identified in the context of H3K4me3 for alcohol dependence (RSM9 and RSM14) were overrepresented for predicted targets of miR-9 (*P* = 2.41E-06).

Overall, our work demonstrates the utility of a systems-biology approach, layering epigenetic and transcriptional co-expression networks, to discern key neighborhoods of genes that are uniquely related to cocaine and alcohol abuse within human brain tissue. Habitual substance abuse is the phenotypic outcome of multiple interacting biological and environmental factors (Farris and Mayfield, 2014), and animal models may only capture a fraction of the underlying molecular and phenotypic variability. Addiction is not caused by any single gene, epigenetic trait, or linear series of additive events. Substance dependence, similar to other relapsing disorders, results from the interplay of multifarious biological occurrences that are propagated through coalescing CNS cellular networks. Integration of diverse “omics” data as functionally related networks provides an infrastructure for grasping the underlying structure of complex phenotypes (Geschwind and Konopka, 2009). Leveraging such network-driven approaches will enable the discovery of novel molecular candidates for detailed investigation in laboratory models. Combined with existing evidence from laboratory studies, networks will permit the rational design of pharmacotherapies for addiction and other debilitating conditions (Hopkins, 2008).

### Conflict of interest statement

The authors declare that the research was conducted in the absence of any commercial or financial relationships that could be construed as a potential conflict of interest.

## Supplementary material

Figure S1 **Weighted gene co-expression networks of RNA-Seq and ChIP-Seq data from postmortem human hippocampus across twenty-three samples**. Shown are dendrograms corresponding to the RNA-Seq data (top) and the H3K4me3 ChIP-Seq data (bottom) based on hierarchical clustering. Modules, colored beneath the dendrograms, were assigned using a dynamic tree cut algorithm to determine the respective groups in each dataset.

Supplemental Table 1 **Differential gene expression of RNA-Seq data for alcohol dependence and cocaine addiction**.

Supplemental Table 2 **Differential expression of H3K4me3 signals (ChIP-Seq) for alcohol dependence and cocaine addiction**.

Supplemental Table 3 **Overrepresented gene ontology groups for WGCNA defined modules from RNA-Seq and ChIP-Seq experiments**.

Supplemental Table 4 **Significantly overlapping RNA-Seq and ChIP-Seq gene modules**.

Supplemental Table 5 **Enrichment of WGCNA gene modules for ChIP-Seq (CSM) and RNA-Seq (RSM) for differentially expressed genes (*P* < 0.05) according to disease status; cocaine addict or alcohol dependence**.

Supplemental Table 6 **WGCNA assignment of gene modules for RNA-Seq and ChIP-Seq experiments**.

Supplemental Table 7 **Significantly overlapping RNA-Seq modules from the current study and superior frontal cortex (CTX) modules from Ponomarev et al. (2012)**.

## References

[B1] Ashburner M. Ball C. A. Blake J. A. Botstein D. Butler H. Cherry J. M.. (2000). Gene Ontology: tool for the unification of biology. Nat. Genet. 25, 25–29. 10.1038/75556 10802651 PMC3037419

[B2] Barski A. Cuddapah S. Cui K. Roh T.-Y. Schones D. E. Wang Z.. (2007). High-resolution profiling of histone methylations in the human genome. Cell 129, 823–837. 10.1016/j.cell.2007.05.009 17512414

[B3] Benayoun B. A. Pollina E. A. Ucar D. Mahmoudi S. Karra K. Wong E. D.. (2014). H3K4me3 breadth is linked to cell identity and transcriptional consistency. Cell 158, 673–688. 10.1016/j.cell.2014.06.027 25083876 PMC4137894

[B4] Bhandari P. Hill J. S. Farris S. P. Costin B. Martin I. Chan C.-L.. (2012). Chloride intracellular channels modulate acute ethanol behaviors in Drosophila, *Caenorhabditis elegans* and mice. Genes Brain Behav. 11, 387–397. 10.1111/j.1601-183X.2012.00765.x 22239914 PMC3527839

[B5] Bierut L. J. (2011). Genetic vulnerability and susceptibility to substance dependence. Neuron 69, 618–627. 10.1016/j.neuron.2011.02.015 21338875 PMC3095110

[B6] Brami-Cherrier K. Lavaur J. Pagès C. Arthur J. S. C. Caboche J. (2007). Glutamate induces histone H3 phosphorylation but not acetylation in striatal neurons: role of mitogen- and stress-activated kinase-1. J. Neurochem. 101, 697–708. 10.1111/j.1471-4159.2006.04352.x 17241117

[B7] Cline M. S. Smoot M. Cerami E. Kuchinsky A. Landys N. Workman C.. (2007). Integration of biological networks and gene expression data using Cytoscape. Nat. Protoc. 2, 2366–2382. 10.1038/nprot.2007.324 17947979 PMC3685583

[B8] Farh K. K.-H. Grimson A. Jan C. Lewis B. P. Johnston W. K. Lim L. P.. (2005). The widespread impact of mammalian microRNAs on mRNA repression and evolution. Science 310, 1817–1821. 10.1126/science.1121158 16308420

[B9] Farris S. P. Arasappan D. Hunicke-Smith S. Harris R. A. Mayfield R. D. (2014). Transcriptome organization for chronic alcohol abuse in human brain. Mol. Psychiatry. 10.1038/mp.2014.159. [Epub ahead of print]. PMC4452464 25450227

[B10] Farris S. P. Mayfield R. D. (2014). RNA-Seq reveals novel transcriptional reorganization in human alcoholic brain. Int. Rev. Neurobiol. 116, 275–300. 10.1016/B978-0-12-801105-8.00011-4 25172479 PMC4267562

[B11] Fienberg A. A. Hiroi N. Mermelstein P. G. Song W. Snyder G. L. Nishi A.. (1998). DARPP-32: regulator of the efficacy of dopaminergic neurotransmission. Science 281, 838–842. 10.1126/science.281.5378.838 9694658

[B12] Geschwind D. H. Konopka G. (2009). Neuroscience in the era of functional genomics and systems biology. Nature 461, 908–915. 10.1038/nature08537 19829370 PMC3645852

[B13] Greengard P. Allen P. B. Nairn A. C. (1999). Beyond the dopamine receptor: the DARPP-32/protein phosphatase-1 cascade. Neuron 23, 435–447. 10.1016/S0896-6273(00)80798-9 10433257

[B14] Griffon N. (2003). CLIC6, a member of the intracellular chloride channel family, interacts with dopamine D2-like receptors. Mol. Brain Res. 117, 47–57. 10.1016/S0169-328X(03)00283-3 14499480

[B15] Guenther M. G. Levine S. S. Boyer L. A. Jaenisch R. Young R. A. (2007). A chromatin landmark and transcription initiation at most promoters in human cells. Cell 130, 77–88. 10.1016/j.cell.2007.05.042 17632057 PMC3200295

[B16] Gupta S. Kim S. Y. Artis S. Molfese D. L. Schumacher A. Sweatt J. D.. (2010). Histone methylation regulates memory formation. J. Neurosci. 30, 3589–3599. 10.1523/JNEUROSCI.3732-09.2010 20219993 PMC2859898

[B17] Harris R. A. Trudell J. R. Mihic S. J. (2008). Ethanol's molecular targets. Sci. Signal. 1, re7. 10.1126/scisignal.128re7 18632551 PMC2671803

[B18] Hawrylycz M. J. Lein E. S. Guillozet-Bongaarts A. L. Shen E. H. Ng L. Miller J. A.. (2012). An anatomically comprehensive atlas of the adult human brain transcriptome. Nature 489, 391–399. 10.1038/nature11405 22996553 PMC4243026

[B19] Hopkins A. L. (2008). Network pharmacology: the next paradigm in drug discovery. Nat. Chem. Biol. 4, 682–690. 10.1038/nchembio.118 18936753

[B20] Hsieh J. Eisch A. J. (2010). Epigenetics, hippocampal neurogenesis, and neuropsychiatric disorders: unraveling the genome to understand the mind. Neurobiol. Dis. 39, 73–84. 10.1016/j.nbd.2010.01.008 20114075 PMC2874108

[B21] Hu S. Xie Z. Onishi A. Yu X. Jiang L. Lin J.. (2009). Profiling the Human protein-DNA interactome reveals ERK2 as a transcriptional repressor of interferon signaling. Cell 139, 610–622. 10.1016/j.cell.2009.08.037 19879846 PMC2774939

[B22] Karlić R. Chung H.-R. Lasserre J. Vlahovicek K. Vingron M. (2010). Histone modification levels are predictive for gene expression. Proc. Natl. Acad. Sci. U.S.A. 107, 2926–2931. 10.1073/pnas.0909344107 20133639 PMC2814872

[B23] Kerns R. T. Ravindranathan A. Hassan S. Cage M. P. York T. Sikela J. M.. (2005). Ethanol-responsive brain region expression networks: implications for behavioral responses to acute ethanol in DBA/2J versus C57BL/6J mice. J. Neurosci. 25, 2255–2266. 10.1523/JNEUROSCI.4372-04.2005 15745951 PMC6726093

[B24] Konopka G. Friedrich T. Davis-Turak J. Winden K. Oldham M. C. Gao F.. (2012). Human-specific transcriptional networks in the brain. Neuron 75, 601–617. 10.1016/j.neuron.2012.05.034 22920253 PMC3645834

[B25] Koob G. F. Volkow N. D. (2009). Neurocircuitry of addiction. Neuropsychopharmacology 35, 217–238. 10.1038/npp.2009.110 19710631 PMC2805560

[B27] Kuhar M. J. Ritz M. C. Boja J. W. (1991). The dopamine hypothesis of the reinforcing properties of cocaine. Trends Neurosci. 14, 299–302. 10.1016/0166-2236(91)90141-G 1719677

[B28] Langfelder P. Horvath S. (2008). WGCNA: an R package for weighted correlation network analysis. BMC Bioinformatics 9:559. 10.1186/1471-2105-9-559 19114008 PMC2631488

[B29] Langfelder P. Zhang B. Horvath S. (2008). Defining clusters from a hierarchical cluster tree: the Dynamic Tree Cut package for R. Bioinformatics 24, 719–720. 10.1093/bioinformatics/btm563 18024473

[B30] Lee Y. J. Shukla S. D. (2007). Histone H3 phosphorylation at serine 10 and serine 28 is mediated by p38 MAPK in rat hepatocytes exposed to ethanol and acetaldehyde. Eur. J. Pharmacol. 573, 29–38. 10.1016/j.ejphar.2007.06.049 17643407 PMC2723821

[B32] Lewohl J. M. Wang L. Miles M. F. Zhang L. Dodd P. R. Harris R. A. (2000). Gene expression in human alcoholism: microarray analysis of frontal cortex. Alcohol. Clin. Exp. Res. 24, 1873–1882. 10.1111/j.1530-0277.2000.tb01993.x 11141048

[B33] Mason M. J. Fan G. Plath K. Zhou Q. Horvath S. (2009). Signed weighted gene co-expression network analysis of transcriptional regulation in murine embryonic stem cells. BMC Genomics. 10:327. 10.1186/1471-2164-10-327 19619308 PMC2727539

[B34] Maze I. Nestler E. J. (2011). The epigenetic landscape of addiction. Ann. N.Y. Acad. Sci. 1216, 99–113. 10.1111/j.1749-6632.2010.05893.x 21272014 PMC3071632

[B35] McClung C. A. Nestler E. J. (2003). Regulation of gene expression and cocaine reward by CREB and Δ FosB. Nat. Neurosci. 6, 1208–1215. 10.1038/nn1143 14566342

[B37] Mulligan M. K. Rhodes J. S. Crabbe J. C. Mayfield R. D. Harris R. A. Ponomarev I. (2011). Molecular profiles of drinking alcohol to intoxication in C57BL/6J mice. Alcohol. Clin. Exp. Res. 35, 659–670. 10.1111/j.1530-0277.2010.01384.x 21223303 PMC3066294

[B38] Nader M. A. Morgan D. Gage H. D. Nader S. H. Calhoun T. L. Buchheimer N.. (2006). PET imaging of dopamine D2 receptors during chronic cocaine self-administration in monkeys. Nat. Neurosci. 9, 1050–1056. 10.1038/nn1737 16829955

[B39] Nestler E. J. (2002). Common molecular and cellular substrates of addiction and memory. Neurobiol. Learn. Mem. 78, 637–647. 10.1006/nlme.2002.4084 12559841

[B40] Pagès H. Lin S. M. Lapointe D. S. Green M. R. (2010). ChIPpeakAnno: a Bioconductor package to annotate ChIP-seq and ChIP-chip data. BMC 11:237 10.1186/1471-2105-11-237 PMC3098059 20459804

[B41] Piechota M. Korostynski M. Solecki W. Gieryk A. Slezak M. Bilecki W.. (2010). The dissection of transcriptional modules regulated by various drugs of abuse in the mouse striatum. Genome Biol. 11:R48. 10.1186/gb-2010-11-5-r48 20459597 PMC2898085

[B42] Pietrzykowski A. Z. Friesen R. M. Martin G. E. Puig S. I. Nowak C. L. Wynne P. M.. (2008). Posttranscriptional regulation of BK channel splice variant stability by miR-9 underlies neuroadaptation to alcohol. Neuron 59, 274–287. 10.1016/j.neuron.2008.05.032 18667155 PMC2714263

[B44] Ponomarev I. Wang S. Zhang L. Harris R. A. Mayfield R. D. (2012). Gene co-expression networks in human brain identify epigenetic modifications in alcohol dependence. J. Neurosci. 32, 1884–1897. 10.1523/JNEUROSCI.3136-11.2012 22302827 PMC3564514

[B52] Ritchie M. E. Phipson B. Wu D. Hu Y. Law C. W. Shi W.. (2015). *Limma* powers differential expression analyses for RNA-sequencing and microarray studies. Nucleic Acids Res. 43:e47. 10.1093/nar/gkv007 25605792 PMC4402510

[B45] Ritz M. C. Lamb R. J. Goldberg S. R. Kuhar M. J. (1987). Cocaine receptors on dopamine transporters are related to self-administration of cocaine. Science 237, 1219–1223. 10.1126/science.2820058 2820058

[B46] Robison A. J. Nestler E. J. (2011). Transcriptional and epigenetic mechanisms of addiction. Nat. Rev. Neurosci. 12, 623–637. 10.1038/nrn3111 21989194 PMC3272277

[B47] Ron D. Messing R. O. (2013). Signaling pathways mediating alcohol effects. Curr. Top. Behav. Neurosci. 13, 87–126. 10.1007/978-3-642-28720-6_161 21877259 PMC3684072

[B48] Sato F. Tsuchiya S. Meltzer S. J. Shimizu K. (2011). MicroRNAs and epigenetics. FEBS J. 278, 1598–1609. 10.1111/j.1742-4658.2011.08089.x 21395977

[B49] Schneider R. Bannister A. J. Myers F. A. Thorne A. W. Crane-Robinson C. Kouzarides T. (2004). Histone H3 lysine 4 methylation patterns in higher eukaryotic genes. Nat. Cell Biol. 6, 73–77. 10.1038/ncb1076 14661024

[B50] Schübeler D. MacAlpine D. M. Scalzo D. Wirbelauer C. Kooperberg C. van Leeuwen F.. (2004). The histone modification pattern of active genes revealed through genome-wide chromatin analysis of a higher eukaryote. Genes Dev. 18, 1263–1271. 10.1101/gad.1198204 15175259 PMC420352

[B43] Sempere L. F. Freemantle S. Pitha-Rowe I. Moss E. Dmitrovsky E. Ambros V. (2004). Expression profiling of mammalian microRNAs uncovers a subset of brain-expressed microRNAs with possible roles in murine and human neuronal differentiation. Genome Biol. 5:R13. 10.1186/gb-2004-5-3-r13 15003116 PMC395763

[B51] Shannon P. Markiel A. Ozier O. Baliga N. S. Wang J. T. Ramage D.. (2003). Cytoscape: a software environment for integrated models of biomolecular interaction networks. Genome Res. 13, 2498–2504. 10.1101/gr.1239303 14597658 PMC403769

[B53] Strahl B. D. Ohba R. Cook R. G. Allis C. D. (1999). Methylation of histone H3 at lysine 4 is highly conserved and correlates with transcriptionally active nuclei in Tetrahymena. Proc. Natl. Acad. Sci. U.S.A. 96, 14967–14972. 10.1073/pnas.96.26.14967 10611321 PMC24756

[B54] Svenningsson P. Nishi A. Fisone G. Girault J.-A. Nairn A. C. Greengard P. (2004). DARPP-32: an integrator of neurotransmission. Annu. Rev. Pharmacol. Toxicol. 44, 269–296. 10.1146/annurev.pharmtox.44.101802.121415 14744247

[B55] Tsai G. Coyle J. T. (1998). The role of glutamatergic neurotransmission in the pathophysiology of alcoholism. Annu. Rev. Med. 49, 173–184. 10.1146/annurev.med.49.1.173 9509257

[B56] Volkow N. D. Wang G.-J. Fowler J. S. Tomasi D. Telang F. (2011). Addiction: beyond dopamine reward circuitry. Proc. Natl. Acad. Sci. U.S.A. 108, 15037–15042. 10.1073/pnas.1010654108 21402948 PMC3174598

[B57] Wang Z. Zang C. Rosenfeld J. A. Schones D. E. Barski A. Cuddapah S.. (2008). Combinatorial patterns of histone acetylations and methylations in the human genome. Nat. Genet. 40, 897–903. 10.1038/ng.154 18552846 PMC2769248

[B58] Zachariou V. Sgambato-Faure V. Sasaki T. Svenningsson P. Berton O. Fienberg A. A.. (2006). Phosphorylation of DARPP-32 at Threonine-34 is required for cocaine action. Neuropsychopharmacology 31, 555–562. 10.1038/sj.npp.1300832 16123776

[B59] Zhang B. Horvath S. (2005). A general framework for weighted gene co-expression network analysis. Stat. Appl. Genet. Mol. Biol. 4:Article17. 10.2202/1544-6115.1128 16646834

[B60] Zhou Z. Yuan Q. Mash D. C. Goldman D. (2011). Substance-specific and shared transcription and epigenetic changes in the human hippocampus chronically exposed to cocaine and alcohol. Proc. Natl. Acad. Sci. U.S.A. 108, 6626–6631. 10.1073/pnas.1018514108 21464311 PMC3081016

