# Supplementary figures and images for: Epigenetic modulation of brain gene networks for cocaine and alcohol abuse
Source: Front Neurosci. 2015 May 20;9:176. doi: 10.3389/fnins.2015.00176 (PMC4438259; doi:10.3389/fnins.2015.00176)

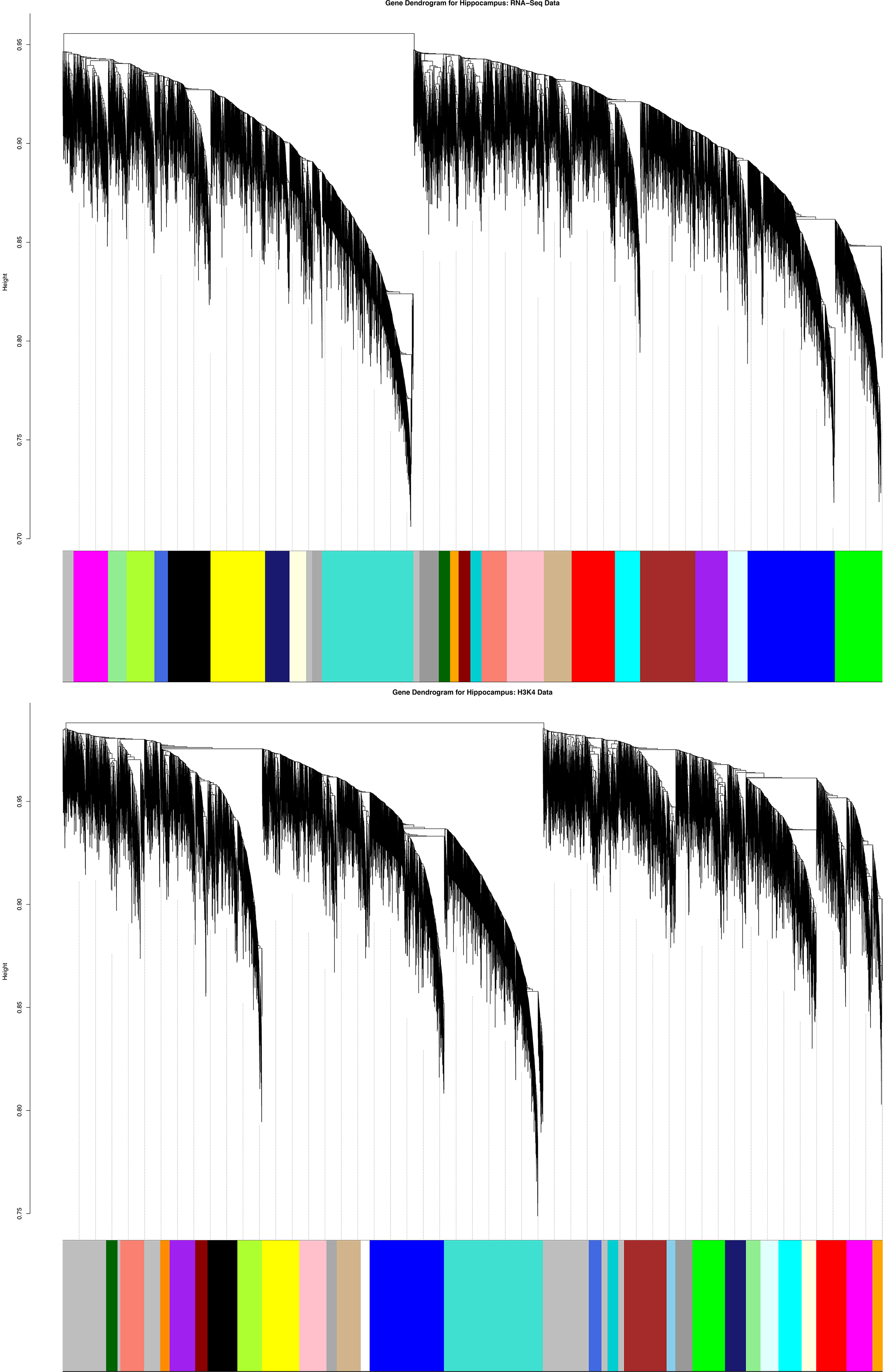

Supplement: Figure S1 — Weighted gene co-expression networks of RNA-Seq and ChIP-Seq data from postmortem human hippocampus across twenty-three samples. Shown are dendrograms corresponding to the RNA-Seq data (top) and the H3K4me3 ChIP-Seq data (bottom) based on hierarchical clustering. Modules, colored beneath the dendrograms, were assigned using a dynamic tree cut algorithm to determine the respective groups in each dataset. [file Image1.TIF]
